# Volatile metabolomic signatures of rabies immunization in two mesocarnivore species

**DOI:** 10.1371/journal.pntd.0007911

**Published:** 2019-12-02

**Authors:** Bruce A. Kimball, Steven F. Volker, Doreen L. Griffin, Shylo R. Johnson, Amy T. Gilbert

**Affiliations:** 1 USDA-APHIS-WS-NWRC, Monell Chemical Senses Center, Philadelphia, Pennsylvania, United States of America; 2 USDA-APHIS-WS-NWRC, Fort Collins, Colorado, United States of America; Universidad Nacional Mayor de San Marcos, PERU

## Abstract

Rabies is a zoonotic disease caused by infection with rabies virus, which circulates naturally in several wild carnivore and bat reservoirs in the United States (US). The most important reservoir in the US from an animal and public health perspective is the raccoon (*Procyon lotor*). To prevent the westward expansion of a significant raccoon rabies epizootic along the eastern seaboard, an operational control program implementing oral rabies vaccination (ORV) has existed in the US since the 1990s. Recently, two vaccine efficacy studies conducted with raccoons and striped skunks (*Mephitis mephitis*) provided the opportunity to determine if volatile fecal metabolites might be used to non-invasively monitor ORV programs and/or predict virus protection for these species. The volatile metabolome is a rich source of information that may significantly contribute to our understanding of disease and infection. Fecal samples were collected at multiple time points from raccoons and striped skunks subjected to oral treatment with rabies vaccine (or sham). Intramuscular challenge with a lethal dose of rabies virus was used to determine protection status at six (raccoons) and 11 (skunks) months post-vaccination. In addition to fecal samples, blood was collected at various time points to permit quantitative assessment of rabies antibody responses arising from immunization. Feces were analyzed by headspace gas chromatography with mass spectrometric detection and the chromatographic responses were grouped according to cluster analysis. Cluster scores were subjected to multivariate analyses of variance (MANOVA) to determine if fecal volatiles may hold a signal of immunization status. Multiple regression was then used to build models of the measured immune responses based on the metabolomic data. MANOVA results identified one cluster associated with protective status of skunks and one cluster associated with protective status of raccoons. Regression models demonstrated considerably greater success in predicting rabies antibody responses in both species. This is the first study to link volatile compounds with measures of adaptive immunity and provides further evidence that the volatile metabolome holds great promise for contributing to our understanding of disease and infections. The volatile metabolome may be an important resource for monitoring rabies immunization in raccoons and striped skunks.

## Author summary

Permanent damage to ecosystems and direct transmission to humans (zoonoses) are two serious consequences of infectious wildlife diseases. Rabies is one such zoonotic disease of concern. Recently, two rabies vaccine efficacy studies were conducted with raccoons (*Procyon lotor*) and striped skunks (*Mephitis mephitis*). These studies allowed us the opportunity to investigate the presence of volatile signals of rabies immunization coded in fecal odors. These signals, if sensitive and specific, could permit non-invasive monitoring of rabies vaccination in wildlife populations. Semi-quantitative analyses of fecal volatiles and quantitative assessment of rabies binding antibodies (rVBA; which represent humoral immune responses to the vaccine or virus) were made at multiple time points. Regression models predicting rVBA responses using volatile fecal metabolites demonstrated predictive qualities in both species. This is the first study to link volatile compounds with adaptive immunity and provides further evidence that the volatile metabolome holds great promise for contributing to our understanding of wildlife diseases.

## Introduction

Rabies is one of the world’s most significant zoonoses, causing an estimated 59,000 cases annually in humans [1]. The most significant wildlife reservoir in the United States (US) is the raccoon (*Procyon lotor*), due in part to their high population densities in suburban and urban habitats [2–4]. Since the 1990s, operational management of wildlife rabies in the US has relied upon oral rabies vaccination (ORV) to control rabies circulation in free-ranging mesocarnivores [5]. While ORV has so far been successful in preventing spread of raccoon rabies west of the Appalachian Ridge in the US, real-time surveillance is needed not only to inform the application of ORV, but also monitor continued intervention success [6].

There is growing evidence that trained animal biosensors may be valuable tools for disease surveillance. Trained detector dogs have already proven to be invaluable wildlife research tools employed for scat [7], carcass [8], and pest detection [9]. Biosensors have been used to discriminate various tissues manifesting cancer from healthy tissue in a number of studies, including lung, prostate, colorectal, ovarian, breast, bladder, and skin cancers [10]. Trained mice have been shown to discriminate between healthy and influenza-infected waterfowl [11] as well as immunization status with an inactivated rabies vaccine in a mouse model [12]. As a result of these and other studies, instrumental investigations of the host’s volatile metabolome as a source of disease signals have recently increased. For example, examination of fecal volatiles by gas chromatography/mass spectrometry has been featured for detection of avian influenza in mallard ducks [11]; bovine tuberculosis in goats [13, 14], white-tailed deer [15], and cattle [16]; and gastrointestinal diseases in humans [17, 18].

The present study takes advantage of vaccination and challenge experiments conducted with raccoons [19] and striped skunks (*Mephitis mephitis*) [20] for the purpose of evaluating oral rabies virus (RABV) vaccine efficacy in these species. The designs of these experiments were particularly useful as they permitted replication in space and time. Given the difficulties associated with containment of viruses and other pathogens, many similar studies have employed small numbers of subjects (often less than 10) and most were only “snapshots” of the volatile metabolome at a single time point. Another strength of the Gilbert et al. studies [19, 20] was the monitoring of the adaptive immune response (RABV neutralizing and binding antibodies, rVNA and rVBA respectively). Although links between cell-mediated immunity and the volatile metabolome have been demonstrated in a mouse model [21, 22], these data provide a unique opportunity to uncover relationships between adaptive immunity and the volatile metabolome. The present study was designed to uncover volatile signatures associated with rabies immunization in two key North American reservoir hosts for RABV.

## Methods and materials

### Ethics statement

Animal use and procedures described here were approved by the USDA National Wildlife Research Center (NWRC) Institutional Animal Care and Use Committee (protocols QA-2258 and QA-2278) and compliant with the animal care and use regulations promulgated in 9 CFR parts 1, 2, and 3.

### Animal subjects

Import and housing of animals at the NWRC facility was permitted under Colorado Parks and Wildlife permits 13TR2056, 14TR2056, 15TR2143, and 16TR2143. Naïve adult and juvenile raccoons and adult striped skunks were obtained from a commercial breeder and housed individually during all phases of the study. All animal procedures summarized below were previously described [19, 20]. Subjects were housed in an Animal Biosafety Level 2 room during challenge and post-infection (pi) monitoring phases. Subjects were fed a daily ration (raccoons 200g; skunks 100g) of Mazuri omnivore diet (PMI Nutrition International, St. Louis, MO) and water was provided *ad libitum*.

Subjects were anesthetized using either 5% isoflurane combined with oxygen or an intramuscular injection of ketamine:xylazine (5:1) for blood sample collection and inoculation. Upon display of two or more clinical signs of rabies, subjects were anesthetized with an intramuscular injection of ketamine:xylazine and euthanized by intracardiac injection of sodium pentobarbital.

### Fecal sample collection

**Fig 1:**
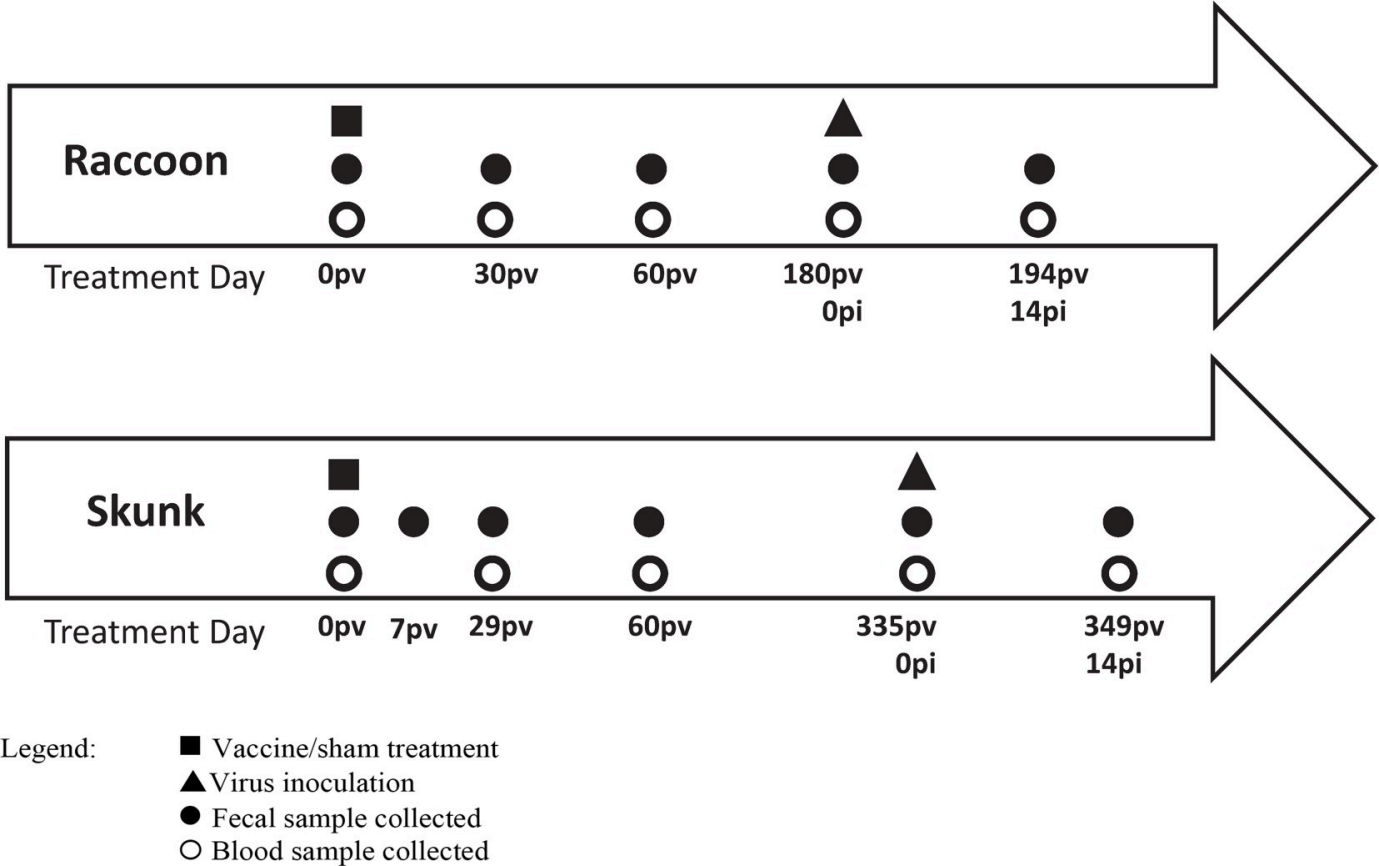
Sample collection schedule at post-vaccination (pv) and post-infection (pi) timepoints for subjects in challenge cohorts (receiving rabies vaccine or sham treatment followed by subsequent challenge with rabies virus).

Animal enclosures were routinely cleaned of all feces at least weekly and additionally the day immediately prior to a planned fecal sample collection. Fecal samples of individually housed animals were collected directly from the pen floor, cage catch pan, or den box. Sample collection occurred while the animal was locked in the associated enclosure den box or otherwise had been temporarily removed from the enclosure for blood sample collection. Samples were collected prior to vaccination and then at pre-determined intervals post-vaccination (pv) and post-infection (pi; Fig 1). On a given collection day, animal enclosures were examined for fresh feces and the freshest appearing were preferentially collected. Approximately 2–5 g was collected directly into a whirl-pak specimen bag. Samples were kept cool on ice packs for up to 6 hrs and then stored at -80 C until analysis.

### Materials

The same vaccine was used in both studies (Ontario Rabies Vaccine, ONRAB; Artemis Technologies, Inc, Guelph, Ontario), but differed in oral delivery format. Importation of the vaccine was authorized by the USDA Center for Veterinary Biologics permit VB-139842. A New York City dog variant of RABV (92-5A), was obtained from the USDA Center for Veterinary Biologics for experimental challenge in both studies as authorized by the USDA Veterinary Services permit 120876. Headspace vials (20-mL) with 18 mm threads, magnetic screw caps with 1.3 mm thickness PTFE/silicone septum, and analytical standard grade (+)-carvone were purchased from Sigma-Aldrich (St. Louis, MO). Solid phase micro extraction (SPME) assemblies with StableFlex (2 cm) divinylbenzene/carboxen/polydimethylsiloxane (DVB/CAR/PDMS) coated fibers were obtained from Supelco Inc. (Bellefonte, PA).

### Raccoons

A total of 21 raccoons (14 male, 7 female) were randomly assigned to one of two treatment groups, live or sham, for presentation of a single ONRAB Ultralite bait during the vaccine efficacy study conducted March through December 2015 (Fig 1). Fifteen raccoons were offered a live vaccine bait containing 1.8mL of vaccine at a titer of 10^9.6^ tissue culture infective doses (TCID50) per mL and six were offered a sham bait during a 24hr presentation window. Blood and fecal samples were collected from subjects prior to vaccination, and then on days 30, 60, 90, and 180 pv.

Seventeen raccoons (four subjects in the vaccine group refused the bait) were challenged on day 180 pv by inoculation with 10^6.2^ MICLD_50_ of the challenge virus (0.5 mL) delivered intramuscularly (IM) into each masseter muscle. Fecal and blood collections were made from surviving animals on day 14 pi. Raccoons were monitored daily up until day 90 pi unless they were euthanized upon display of two or more clinical signs of rabies. Surviving raccoons were euthanized on or after day 90 pi. Rabies was diagnosed from brainstem and cerebellar tissues collected postmortem from all subjects by direct fluorescent antibody assay (DFA) at the Colorado State University Veterinary Diagnostic Laboratory.

### Skunks

A total of 20 skunks (12 male, 8 female) were randomly assigned to one of three vaccine groups or control (n = 5 each) for use in a vaccine efficacy study conducted July 2014 through June 2015. The three vaccine groups represented three doses (10^10.2^, 10^9.8^, or 10^9.3^ TCID50) delivered in minimal essential media (MEM) supplemented with 5% fetal bovine serum. The control group received MEM only. Vaccine or MEM were delivered by DIOC (direct instillation into the oral cavity) route under light anesthesia. Blood and fecal samples were collected from subjects prior to vaccination, and then on days 29, 60, 90, and 335 pv. Feces (only) were also collected on day 7 pv (Fig 1).

All skunks were challenged with the 92-5A challenge virus on day 335 pv as previously described. Blood and fecal samples were collected from surviving animals on day 14 pi. Skunks were monitored daily up until day 75 pi unless they were euthanized upon display of two or more clinical signs of rabies. Surviving skunks were euthanized on or after day 75 pi. Rabies was diagnosed from brainstem and cerebellar tissues collected postmortem from all subjects as described above for raccoons.

### Detection of rabies virus antibodies

Serum samples were assessed for RABV neutralizing antibodies (rVNA) analyses by rapid fluorescent focus inhibition test (RFFIT) at Kansas State University (KSU) as previously described [19]. Samples with greater than or equal to 0.1 international units per milliliter (IU/mL) were considered seropositive. Samples reported as less than 0.1 IU/mL were considered negative for rVNA and were recoded to have a value of 0.05 IU/mL for the purpose of statistical evaluation.

A subset of raccoon blood samples was tested for RABV binding antibodies (rVBA) at KSU using a commercial indirect ELISA (BioRad Platelia Rabies Kit II, Marnes-la-Coquette, France). All reported and predicted rVBA values less than 0.125 (negative) were assigned a value of 0.0625 equivalent units per mL (EU/mL) for the purpose of statistical evaluation.

A subset of skunk blood samples was tested for rVBA at NWRC using a commercial blocking ELISA (BioPro Rabies ELISA, OK Servis, Prague, Czech Republic) following the manufacturer’s instructions. A sample was considered positive for rVBA by ELISA if the percent blocking was equal to or greater than 40% per manufacturer instructions. Values less than zero were recoded as zero for statistical analyses.

### Volatile fecal metabolome analyses

One gram of feces (raccoon or skunk) was individually placed in 20-mL glass headspace vials, capped, and refrigerated until analysis. Immediately prior to analysis, vials and contents were warmed to room temperature and the internal standard (0.010 mL of 70 ppm (S)-carvone prepared in water) was added using a syringe. Headspace extraction was performed with a PAL autosampler (Agilent Technologies, Santa Clara, CA) equipped with a SPME fiber assembly (DVB/CAR/PDMS). The sample was pre-incubated for 10 min at 37°C with pulsed agitation (250 RPM for 5 s, off for 2 s) and the SPME needle was inserted into the vial for 40 min headspace collection. Collected volatiles were desorbed from the SPME fiber at 270 ^o^C for 1 min in the injection port of an Agilent 7890B gas chromatograph (Agilent Technologies, Santa Clara, CA) equipped with an ultra-inert straight liner and 23 ga. Merlin Microseal septum (Merlin Instrument Co., Newark, DE). Chromatographic separation was achieved using a 30 m x 0.25 mm ID Stabilwax-DA (0.25μm film thickness) capillary column (Restek Corp., Bellefonte, PA) with helium carrier gas in constant flow mode (1.0 mL/min). The oven temperature was held at 35°C for 2.5 min, increased at 6.0°C/min to 260°C, and then held for 5 min. The gas chromatograph was coupled to an Agilent 5977A mass selective detector (MSD) for collection of electron impact (EI) spectra over the range of 50 to 500 m/z. Empty vials and empty vials fortified with carvone were also analyzed. Mass spectral peak identifications were assigned based on the library search of the NIST Standard Reference Database.

Although instrumental analyses of samples from the separate raccoon and skunk studies were not conducted concurrently, all chromatograms were processed simultaneously. Chromatographic data were exported to mzData format using Proteowizard software [23] followed by baseline correction, noise elimination, and peak alignment using Metalign^TM^ software [24]. The processed data (consisting of all mass spectrometric responses exceeding a defined threshold at each scan event) were subjected to unsupervised peak identification and integration using the MSClust tool [25]. The resultant data set (consisting of a single response for each peak identified in the chromatogram) was subjected to principal components analysis (PCA) using Unscrambler (CAMO Software; Oslo, Norway) to visually identify sample analysis outliers exhibiting undue influence or leverage in residual plots. Prior to statistical modelling, all peak responses were standardized according to sample mass and S-carvone peak response observed in the sample. Peaks not attributed to feces (e.g. peaks observed in empty vials) were dropped from the data set.

### Data analyses

The VARCLUS procedure in SAS was used to group square root transformed carvone-standardized peak responses into related clusters and scores were calculated using the SCORE procedure. Clustering was performed separately for each species. Multivariate analyses of variance (MANOVA) were conducted for the post-vaccination period through day 60 pv (not including day 0 pv) (Fig 1). Together, the cluster score responses were termed “volatiles” for within-subjects tests. Survival (as the measure of viral protection) and experiment day were considered fixed effects. Subject (nested in survival) was a random effect. Residuals were evaluated for normality and homoscedasticity and outlier observations (based on residual plots) were removed.

The relationships between immune responses (rVNA or rVBA) and individual components of the volatile fecal metabolome were evaluated by stepwise multiple regression model building using the REG procedure in SAS. All samples corresponding to days 0 pv through day 14 pi from subjects receiving vaccine treatment with measured immune responses were included in model building (Table 1). Separate models were built for each species (raccoon, skunk) and immune response (rVNA, rVBA). Residuals were evaluated for normality and homoscedasticity and outlier observations (based on residual plots) were removed. Models for rVNA required square root transformation of the rVNA response. Correlations (predicted vs. measured) were calculated for model samples using the CORR procedure in SAS.

**Table 1:** Timeline and quantity of sera samples from vaccinated subjects used for multiple regression model building for rabies neutralizing antibody (rVNA) and rabies binding antibody (rVBA) responses. Experimental days reflect post-vaccination (pv) or post-infection (pi).

	Days	rVBA Samples	rVNA Samples
Raccoon	0 pv, 30 pv, 60 pv, 0 pi, 14 pi	42	52
Skunk	0 pv, 29 pv, 60 pv, 0 pi, 14 pi	67	68

The likelihood that a linear relationship between immune response and volatile metabolome could arise from a random data set (potential type I error) was estimated for all four multiple regression models (rVNA and rVBA for both skunks and raccoons). For each model, 1000 unique data sets comprised of 50 randomly generated peak responses and the actual measured values for the immune responses were created. All data sets were subjected to stepwise multiple regression building and the number of models (out of 1000) resulting in R^2^ exceeding that of the actual model (using the same number of predictors, or less) was recorded.

## Results

### Vaccination and challenge

Efficacy results of the vaccination studies with raccoons and skunks have previously been reported [19, 20]. Summarized results below are specific to the subjects sampled for fecal volatiles.

### Raccoon

Peak rVNA and rVBA response among vaccinated animals (prior to challenge) was observed around day 60 pv. Among sham-baited animals, neither rVNA nor rVBA were detected at any time point prior to challenge. Survival following lethal RABV challenge was 73% (8 of 11) among vaccinates. Because a small number of vaccinated animals succumbed to viral challenge, survival was considered a more appropriate measure of virus protection as compared to vaccine treatment. All six control (sham-vaccinated) raccoons succumbed to rabies challenge. All mortalities occurred by day 14 pi and tested positive for rabies by DFA test.

### Skunk

Vaccination induced robust rVNA titers in all but a single animal vaccinated by direct instillation (this subject did not survive virus challenge). No sham-treated animals developed rVNA at any pre-challenge time point. Peak rVNA and rVBA were observed between days 29 pv and 60 pv for each vaccine dose tested. One vaccinated subject died prior to challenge and was removed from the study. Across all vaccinates, survival following lethal RABV challenge was 93% (13 of 14). Five control (sham-vaccinated) skunks developed rabies. Three of six mortalities occurred prior to day 14 pi, while three others occurred on days 16, 19, or 33 pi. All mortalities were confirmed rabies positive by DFA test.

### Volatile fecal metabolome

Principal components analysis identified one sample (from a raccoon) considered to be an outlier and was removed from the data set. Fifty-two unique chromatographic peaks arising from feces (including the carvone standard) were identified in the headspace. One peak was identified as isoflurane (anesthetic used during these studies), which was not included in any data analyses. For both raccoons and skunks, the 50 carvone-standardized peak responses were grouped into 12 clusters. Although clustering was performed separately, there was considerable grouping similarity between the species. For example, free fatty acids tended to cluster together for both species as did many aldehydes and exogenous compounds (e.g, xylenes).

### Raccoon MANOVA

Experiment day (p = 0.0026) was the only significant between-subjects effect. The significant within-subjects interaction of volatiles and survival (p = 0.006) indicated that some clusters were associated with protection status of raccoons. Univariate results identified a significant individual cluster having methyl isobutyl ketone, toluene, and 1-octen-3-ol as its members. The volatiles*survival*experiment day interaction was not significant, suggesting that this cluster differed by survival but not experiment day. Inspection of the individual volatiles in this cluster indicated that the square root responses of each of the three compounds were lower at days 30 and 60 pv in subjects that ultimately survived the virus.

### Raccoon immune response model

Two extreme rVNA responses were identified and square root transformation of rVNA responses was necessary to ensure normality and homoscedasticity of residuals. The multiple regression model for rVNA using five chromatographic peak response predictors was significant (p = 0.0014) with an R^2^ = 0.352. The probability that this model could arise randomly was high, with 736 of 1000 random models explaining at least the same variation using nine or fewer predictors (p = 0.736).

**Fig 2:**
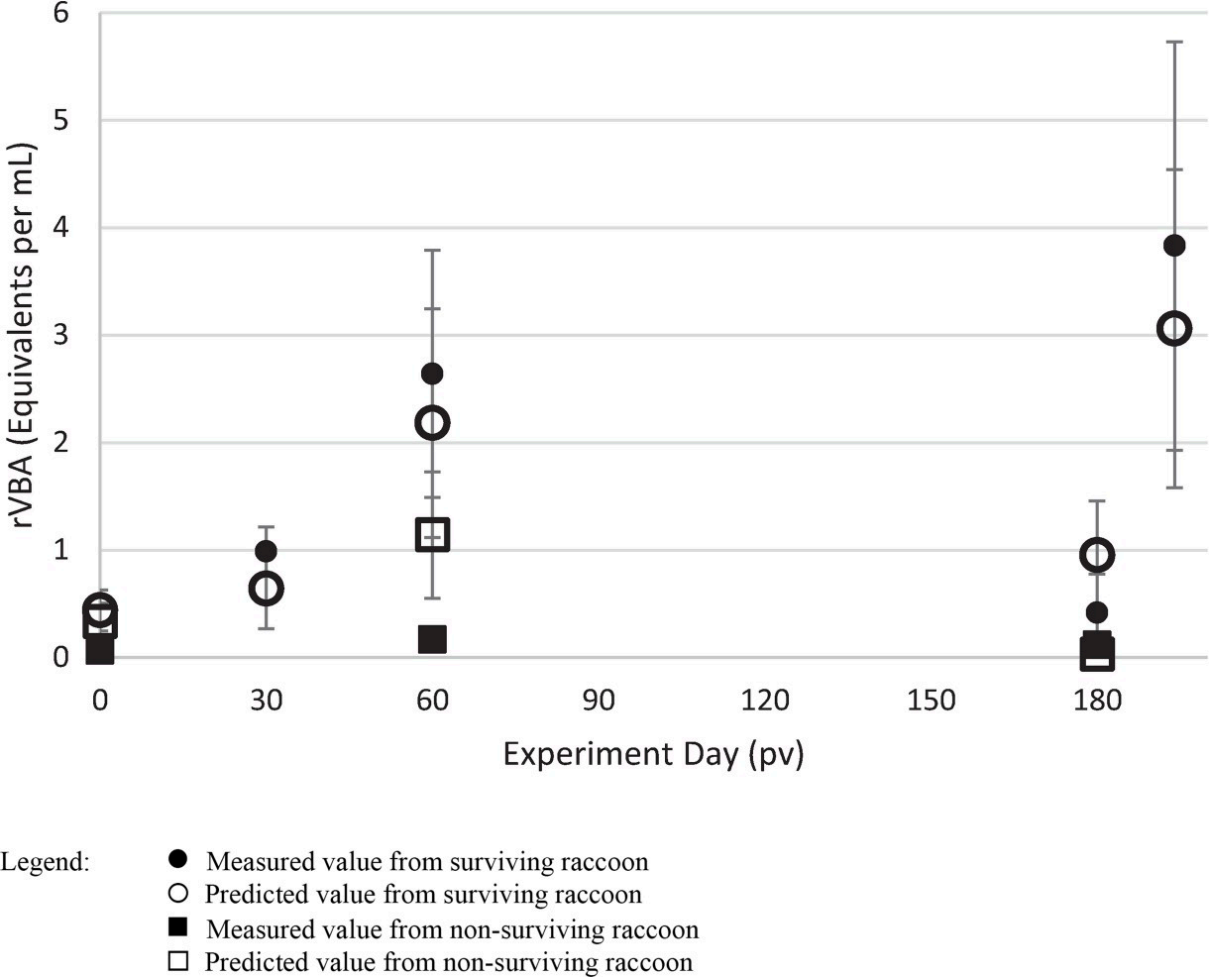
Measured and predicted rabies binding antibody values (rVBA) in raccoons at post-vaccination (pv). Infection occurred on day 180 pv. Error bars represent standard errors.

The rVBA model did not require transformation of the responses, although two extreme outliers were identified and removed from any further consideration. The resulting multiple regression model (p < 0.0001) employed 7 predictors (Table 2) and yielded an R^2^ = 0.792. The models for rVNA and rVBA shared only one common predictor (2-ethyl-1-hexanol). Evaluation of the random data sets indicated a very low probability for this model arising at random (p = 0.03). Predicted values less than zero were recoded as zero and one predicted rVBA response greatly exceeded the range of measured responses and was removed from further consideration. Predicted rVBA responses were in excellent agreement with measured values (Fig 2A; r = 0.903; p < 0.0001).

**Table 2:** Tentative compound identifications for chromatographic peaks belonging to volatile clusters associated vaccination (CLUS) or immune regression model (rVBA) where symbol identifies positive regression coefficients (+) or negative regression coefficients (-).

Compound Name	Raccoon		Skunk	
	CLUS	rVBA	CLUS	rVBA
Benzaldehyde				+
2-Butyl-2-octenal		**-**		
2-Ethylhexanoic acid		**-**		
2-Ethyl-1-hexanol		**+**		
Hexanal			X	
Isobutyric acid		**+**		
Methylisobutylketone	X			
2-Methylquinoline (also Quinaldine)				+
4-Nonanone			X	-
γ-Nonalactone				+
1-Octen-3-ol	X			+
2-Pentylfuran			X	
Phenol				-
Propanoic acid		**-**		
Propanoic acid, 2-methyl-2,2-dimethyl-1-(2-hydroxy-1-methylethyl)propyl ester		**-**		-
Toluene	X			
Unknown compound A			X	+
Unknown compound B		**+**		

### Skunk MANOVA

Identical to the raccoon data, experiment day (p = 0.0298) and volatiles*survival (p = 0.0237) were the only significant effects. Also like the raccoon data, one cluster differed by survival over the post-vaccination period (days 7, 29, and 60 pv). The compounds hexanal, 2-pentylfuran, 4-nonanone, and an unknown were members of this cluster. Inspection of the individual compounds did not reveal anything remarkable about their square root responses as they related to survival.

### Skunk immune response model

**Fig 3:**
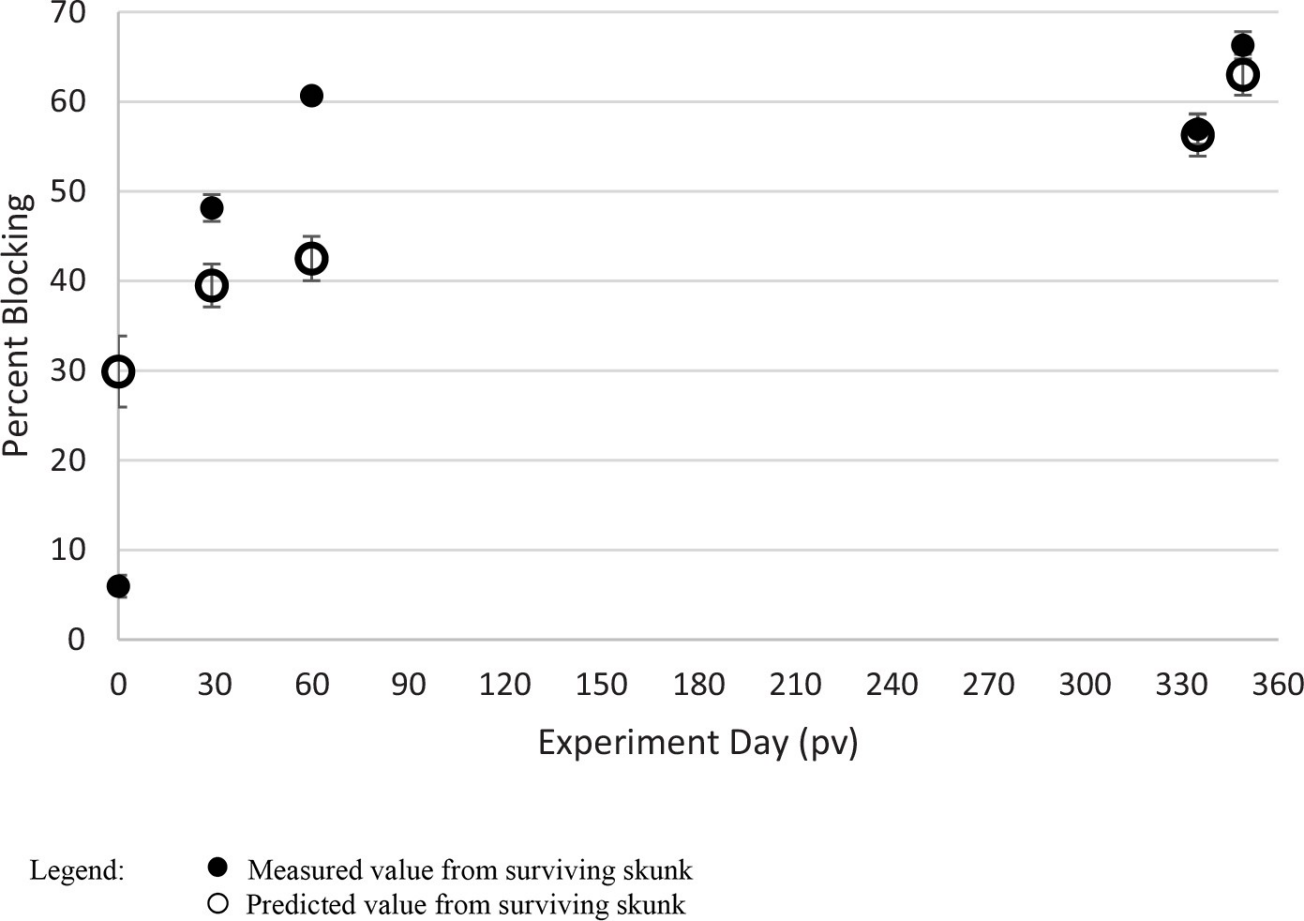
Measured and predicted rabies binding antibody values (rVBA) in skunks at post-vaccination (pv). Infection occurred on day 335 pv. Only one skunk from the model set died during virus challenge and was not included in the figure. Error bars represent standard errors.

None of the 68 rVNA values were identified as outliers, but similar to the raccoon data, square root transformation of the responses was required. Also similar to the raccoon model, the model for rVBA explained greater variation (R^2^ = 0.443 using eight predictors) as compared to the model for rVNA (R^2^ = 0.1402 using three predictors). The rVNA and rVBA models shared one predictor (1-octen-3-ol). Estimates of type I errors for these models further demonstrated the very poor quality of the rVNA model (p = 0.838) and the relatively high probability that the rVBA model could also arise from random data (p = 0.429). Predicted rVBA responses were in good agreement with measured values, but the correlation of skunks was lower than raccoons (Fig 3; r = 0.665; p < 0.0001).

## Discussion

The observation that three vaccinated raccoons and one vaccinated skunk succumbed to rabies upon virus challenge was not unprecedented [26]. For this reason, survival (rather than vaccine treatment) was considered a design effect. Moreover, prediction of survival better fits the objective of this metabolomic study to develop a tool for monitoring population health. The MANOVA design applied here for examination of the volatile metabolome was meant to uncover volatiles that varied according to vaccine protection (i.e. significant within-subjects survival*volatiles effect). A significant survival*volatiles*experiment day would be undesirable as it would indicate that volatiles related to virus protection differed over time.

While the raccoon and skunk experiments provided a unique opportunity to examine the volatile metabolome across an extended protected period, introduction of this source of variation (i.e. measurement at multiple timepoints) demonstrates the difficult nature of identifying robust biomarkers of disease. This design feature is in contrast to the majority of previous studies of animal health and fecal volatiles where measurements were made at a single time point representing the healthy status and/or the disease state. Such “snapshots” have been used in many recent studies to identify disease or infection using volatile metabolites present in feces [11, 14–16], breath [14, 27–30], or other emanation [31–33]. To be useful for monitoring population health, characterized signals must be robust and reliable over extended periods, not just at specific time points following immunization or infection. This study suggests that the variability of the volatile metabolome may be too high over time and space to permit identification of an underlying and specific signal of vaccine-induced protection from rabies virus infection.

The prospect of exploiting the volatile metabolome for disease diagnosis arises from decades (if not centuries) of anecdotal and empirical evidence [34]. This promise follows logically from an understanding of chemical communication and the adaptive advantage afforded by a system that advertises the health status of conspecifics. Accordingly, animal biosensors have convincingly demonstrated that disease diagnoses may be made on the basis of odor alteration resulting from disease or infection [35–37] and are capable of detecting underlying volatile signals in the face of dietary variation [38]. Although chemometric data collected from analyses of bodily fluids (such as feces) or other emanations (e.g. breath) have yielded explanatory models that discriminate between healthy and infected subjects in many disease systems in [39–42], a chemometric approach based on the analyses of individual odorants may not capture the same information available to a trained animal biosensor. Alternatively, a critical signal of immunization may be more robust in the days immediately following delivery of the vaccine. In a study in the mouse model, a rabies vaccination signal was evident to trained biosensors evaluating urines collected from days 4 to 13 pv [12]. However, later time points were not examined in that study. The sampling design in this study was guided by the timeframe during which post-ORV monitoring (i.e., live animal trapping and blood collection) is performed in the US, which is typically 4–6 weeks following baiting. As more and more experiments are conducted, it has become evident that virtually any perturbation of host health will result in alteration of its volatile metabolome [43].

Although the MANOVA models did not yield highly informative indicators of protection status, the regression models constructed for rVBA responses demonstrated excellent capacity to quantify the immune response to the vaccine, particularly in raccoons. It is was unsurprising that the metabolomic predictors chosen for the rVBA and rVNA models differed. Immune responses to vaccination, including rabies vaccines, are related to the major histocompatibility complex (MHC) [26]. Genetic variability in MHC as small as one base pair results in detectable differences in the volatile metabolites of in-bred mice [44]. However, it was surprising that the rVBA models for both species were superior to the corresponding rVNA models. This was also reflected by the fact that measured rVBA data demonstrated more reliable survival predictions for experimentally-challenged carnivores as compared to rVNA [26]. Differences between these results may be due, in part, to the greater variability of rVNA due to employment of live virus and cell culture in serum neutralization tests. In other words, determination of rVBA is a direct measurement of the immune response (i.e. antibody), while rVNA is an indirect measure of function. Thus, a direct link between the rVBA measurement and some aspect of the volatile metabolome may also exist when an association with rVNA measurement does not.

Tentative identifications for specific metabolites identified in this study suggest that many of the volatile fecal metabolites associated with protection from rabies virus are under the control of cellular fatty acid metabolism (Table 3). Nutrients such as glucose, amino acids, and fatty acids serve as the fuel supporting metabolically costly immune activation [45]. For example, among the humoral responses of immunization is the production of T cells whose regulation relies on fatty acid metabolism [46]. Products of cell-mediated immune responses (i.e. cytokines) have also been tied to alterations of the volatile metabolome [22]. Thus, alterations of the volatile metabolome may reflect these metabolic costs.

**Table 3:** Natural origin of metabolites associated with rabies volatile fecal metabolome.

Compound Name	Fatty Acid Metabolism^1^	Food^1^	GI^2^	Micro^3^	Synth/ Enviro^4^
Benzaldehyde		X	X		
2-Butyl-2-octenal		X			
2-Ethylhexanoic acid	X	X			X
2-Ethyl-1-hexanol	X	X			
Hexanal		X	X		
Isobutyric acid	X	X	X	X	
Methyl isobutyl ketone		X	X		X
2-Methylquinoline		X			
4-Nonanone		X			
γ-Nonalactone		X			
1-Octen-3-ol		X	X		
2-Pentylfuran		X	X		
Phenol		X	X		X
Propanoic acid	X	X	X	X	
Propanoic acid, 2-methyl-2,2-dimethyl-1-(2-hydroxy-1-methylethyl)propyl ester					X^5^
Toluene		X	X		X
Unknown compound A					
Unknown compound B					

^1^Endogenous source according to HMBD 4.0 [48]

^2^Human fecal metabolites associated with gastrointestinal diseases [17]

^3^Microbiological source according to HMBD 4.0 [48]

^4^Industrial application according to HMBD 4.0 [48]

^5^Family of plasticizers

Nearly all the identified volatile metabolites have a dietary source and some are known to be metabolites of bacterial metabolism and/or human gastrointestinal diseases (Table 3). Thus, the gut microbiome may have an important role in regulation of the volatile metabolome associated with immunization and/or infection. For example, the gut microbiome has been implicated as significant contributor of increased acetoin concentrations in feces of waterfowl infected with avian influenza [11]. Integration of host and microbiome metabolism may be crucial to understanding mechanisms governing alterations of the volatile metabolome [43].

However, a few of the predictors are non-endogenous and/or have non-food environmental sources (Table 3). Although the studies with raccoons and striped skunks were conducted at different times, both species were held in the same facility and were fed the same diet. The importance of both food-source and possible environmental contaminants in the discriminant models suggest that absorption, biotransformation, and excretion processes are also involved–raising the possibility that the volatile fecal metabolites identified in chemometric studies may vary regionally and/or seasonally.

It is evident that the volatile metabolome is a rich source of information that holds great promise for contributing to our understanding of diseases in humans and wildlife. Studies using instrumental approaches to evaluate the volatile metabolome have successfully demonstrated the explanatory capabilities of discriminant models in many disparate systems of species and pathogens. Great care should be made toward understanding that excellent explanatory models may not be highly predictive.

A tool for predicting rVBA responses non-invasively may be useful for monitoring the health of wild populations susceptible to rabies. Currently, serology tools to monitor ORV in the US require animal capture for biological sampling [5]. Bait markers are also used to monitor vaccine exposure and evaluate bait uptake. However, identification of an optimal marker compound for this purpose can be challenging. For example, analysis of tetracycline deposition in the teeth of animals that have ingested ORV baits is intrusive, time consuming, and requires animal anesthesia [5]. Alternatively, fecal analyses do not require capture and handling of animals, and could be a useful noninvasive sample [47]. This study suggests that rabies antibody responses may be reliably predicted via analysis of the volatile metabolome in fecal samples. However, several procedural evaluations are required before an ORV monitoring program consisting of fecal collection and volatile analyses can be implemented. First, the age and condition of the feces are likely to influence the volatile metabolome (the current study enjoyed the advantage of fresh sample collection and animals consuming a standardized diet). Second, rVBA predictions appeared superior in raccoons versus skunks. It must be determined whether these differences can be attributed to species, the kit(s) used, or the laboratory which performed the assays. Importantly, these are not insurmountable. If properly addressed, the volatile metabolome may serve as an effective means to monitor animal health.
